# Primary extranodal soft tissue Lennert lymphoma (lymphoepithelioid variant of peripheral T-cell lymphoma, unspecified): a case report and review of the literature

**DOI:** 10.1186/s13000-023-01297-w

**Published:** 2023-02-03

**Authors:** Ying Yin, Huaipu Liu, Minghua Luo, Guangyin Yu, Weihua Yin, Ping Li

**Affiliations:** 1 Department of Pathology, Peking University Shenzhen Hospital, 1120 Lianhua Road, Shenzhen, 518036 Guangdong Province China; 2 Department of Cardiothoracic Surgery, Shenzhen Children’s Hospital, 7019 Yitian Road, Shenzhen, 518038 Guangdong Province China

**Keywords:** Peripheral T-cell lymphoma, Lennert type, Lennert lymphoma, Soft tissue

## Abstract

Lennert lymphoma (LeL) is a rare variant of peripheral T-cell lymphoma, not otherwise specified (PTCL/NOS) that is rich in epithelioid histiocytes. LeL may pose great diagnostic and therapeutic challenges to the pathologist and clinician. Primary extranodal soft tissue LeL is even rarer and has not been reported. Herein, we report a case of LeL arising from soft tissue.

A 65-year-old male presented for evaluation of a painless mass in the subcutaneous soft tissue of the left forehead. There was no invasion of the bone and no ulceration on the surface of the skin. The surrounding skin was erythematous and swollen. Grossly, the tumor was gray-red and 30 mm × 20 mm × 10 mm in size.

Microscopically, the demarcation between the lesion and surrounding tissues was unclear without a capsule. The tumor invaded the surrounding striated muscle and adipose tissue. The tumor had a diffuse proliferation of small-sized atypical lymphocytes and numerous large clusters of epithelioid histiocytes. Plasma cells, eosinophils, and Hodgkin-Reed-Sternberg (HRS) cells were not identified. Rare multinucleated histiocytes were noted, and well-formed granulomas were not present. Rare mitotic figures were noted, but no necrosis. The immunophenotypic features in this case were as follows: CD2^+^/CD3^+^/CD5^low+^/CD7^+^/CD4^low+^/ CD8^+^/CD30^−^/CD56^−^ in neoplastic lymphocytes; CD163^+^/CD31^+^/CK(pan)^−^ in epithelioid histiocytes; and CD20^−^/CD30^−^/TdT^−^/CD5^−^/ALK^−^/S-100^−^/CD1α^−^/CD21 + 23^−^/SSTR2^−^ in neoplastic lymphocytes and epithelioid histiocytes. Epstein-Barr virus (EBV)-encoded RNA in situ hybridization (EBER-ISH) was negative. The Ki-67 index was elevated to 60%. PCR showed a polyclonal pattern for IgH and a monoclonal TCR γ-chain rearrangement.

The final diagnosis was PTCL/NOS, lymphoepithelioid cell variant (LeL), which arose from soft tissue and had a rare double-positive CD4^low+^/CD8^+^ immunophenotype. The patient received four cycles of cyclophosphamide, doxorubicin liposomes, vincristine, and prednisone tablets (CHOP) and was followed for 20 months. Overall treatment efficacy was achieved without lymphadenopathy, and no other discomfort or illnesses were reported.

## Introduction

Lennert lymphoma (LeL) is classified as a “lymphoepithelioid cell variant of the peripheral T-cell lymphoma, not otherwise specified (PTCL/NOS)” according to the World Health Organization classification [1, 2]. LeL is a rare morphologic variant of a peripheral T-cell lymphoma that is characterized by epithelioid histiocyte proliferation without formation of discrete granulomas admixed with small-sized atypical lymphocytes [3, 4]. Etebari et al. [5] determined the transcriptional profile of 12 LeL cases and concluded that LeL is a distinct entity rather than simply a morphologic variant of PTCL/NOS. This view is consistent with the findings of Joe et al. [6]. Because of the special morphologic features, LeL has a high rate of misdiagnoses and missed diagnoses. Genetic studies remain important adjuncts for demonstrating T-cell receptor (TCR) gene rearrangements as a marker of T-cell clonality, especially in difficult cases.

Herein we report a case of primary extranodal soft tissue LeL (lymphoepithelioid variant of PTCL/NOS) in a 65-year-old man with a rare double-positive CD4^low+^/CD8^+^ immunophenotype. Such a case is extremely rare and has not been reported in the literature.

## Case report

The patient was a 65-year-old male who presented for evaluation of a painless mass in the subcutaneous soft tissue of the left forehead. There was no invasion of the bone and no ulceration on the surface of the skin. The surrounding skin was erythematous and swollen. There were no special clinical manifestations or laboratory test abnormalities. Grossly, the tumor was gray-red and 30 mm × 20 mm × 10 mm in size. There was no pertinent family history.

**Fig. 1:**
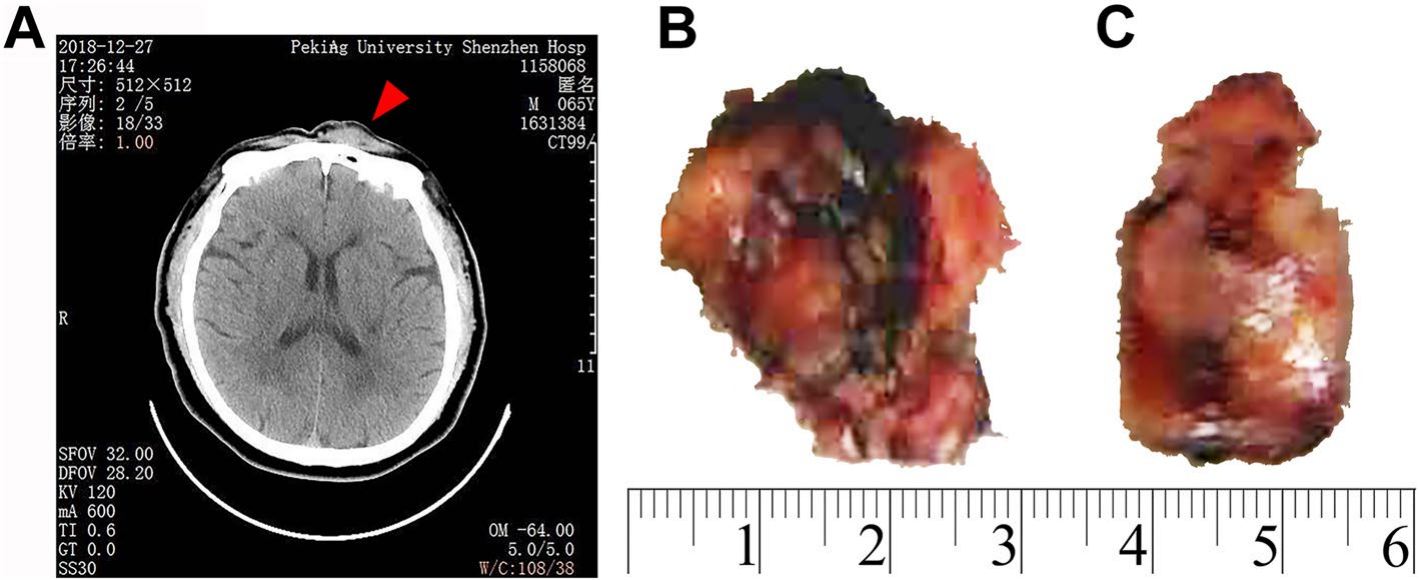
**A** Plain CT scan of the brain showed swelling of the subcutaneous soft tissue of the forehead with increased density and CT value of 63 HU. There was no invasion of the bone. **B** Grossly, the tumor was gray-red and 30 mm × 20 mm × 10 mm in size. **C** The cut surface of the mass was solid, grey-red, and slightly firm

A plain CT scan (Fig. 1A) of the brain showed swelling of the subcutaneous soft tissue of the forehead with increased density and a CT value of 63 HU. The largest area of swelling was 31 mm × 9 mm. Skull size, shape, and density were normal based on CT. There was no bony invasion. The frontal mass was surgically removed. The tumor was gray-red and 30 mm × 20 mm × 10 mm in size on gross pathologic examination (Fig. 1B). The cut surface of the mass was solid, grey-red, and slightly firm (Fig. 1C).

**Fig. 2:**
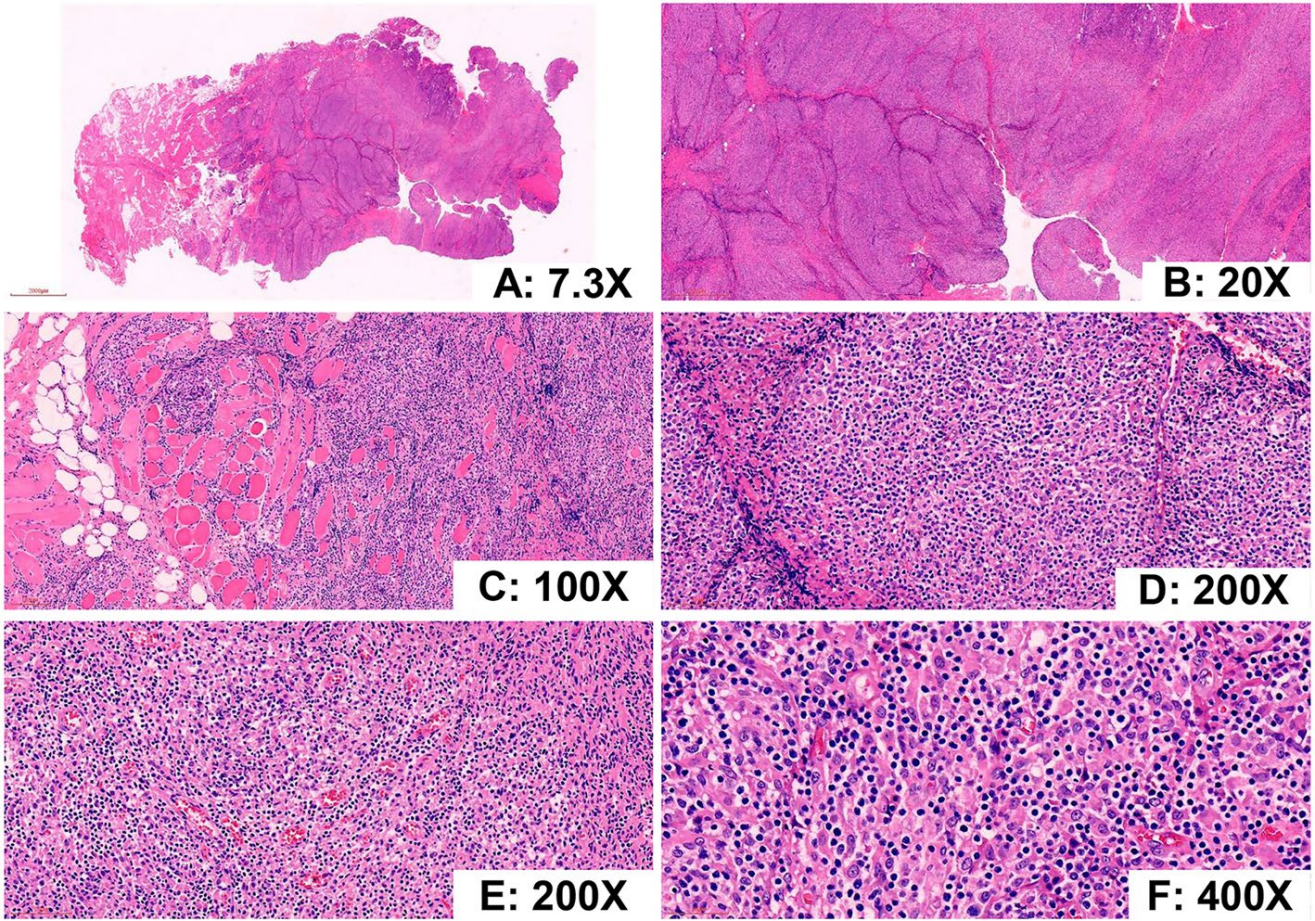
Microscopic findings (Haematoxylin and eosin [HE] staining). **A** The demarcation between the lesion and surrounding tissues was unclear and lacked a capsule. **B** The lesion appeared multinodular in shape. Nodules were surrounded by proliferative fibrous connective tissue. **C** The tumor invaded the surrounding striated muscle and adipose tissue. **D**, **E** The tumor showing a diffuse proliferation of small lymphocytes and numerous large clusters of epithelioid histiocytes. **F** At high magnification, the proliferation of small lymphocytes displayed slight nuclear irregularities. Plasma cells, eosinophils, and Hodgkin-Reed-Sternberg (HRS) cells were not identified. Rare multinucleated histiocytes were noted, and well-formed granulomas were not present. Rare mitotic figures and no necrosis were observed. (HE staining, **A** 7.3×; **B** 20×; **C** 100×; **D** 200×; **E** 200×; **F** 400×)

At low magnification, the demarcation between the lesion and surrounding tissues was unclear and lacked a capsule (Fig. 2A). The lesion appeared multinodular in shape, and the nodules were surrounded by proliferative fibrous connective tissue (Fig. 2B). Figure 2C shows that the tumor invaded the surrounding striated muscle and adipose tissue. On medium magnification (Fig. 2D, E) a diffuse proliferation of small lymphocytes and numerous large clusters of epithelioid histiocytes were noted. On high magnification (Fig. 2F) the proliferation of small lymphocytes displayed slight nuclear irregularities with scanty cytoplasm and hyperchromatic nuclei. The cell nuclei were round and oval, localized at or near the center of the cell body, and the nucleoli were small. These cells showed mild atypia. No plasma cells, eosinophils, or Hodgkin-Reed-Sternberg (HRS) cells were noted. Rare multinucleated histiocytes were present, but no well-formed granulomas were apparent. Rare mitotic figures were seen, but no necrosis was observed. Small blood vessels were evenly distributed in the interstitium, but endothelial cell proliferation was not significant.

**Fig. 3:**
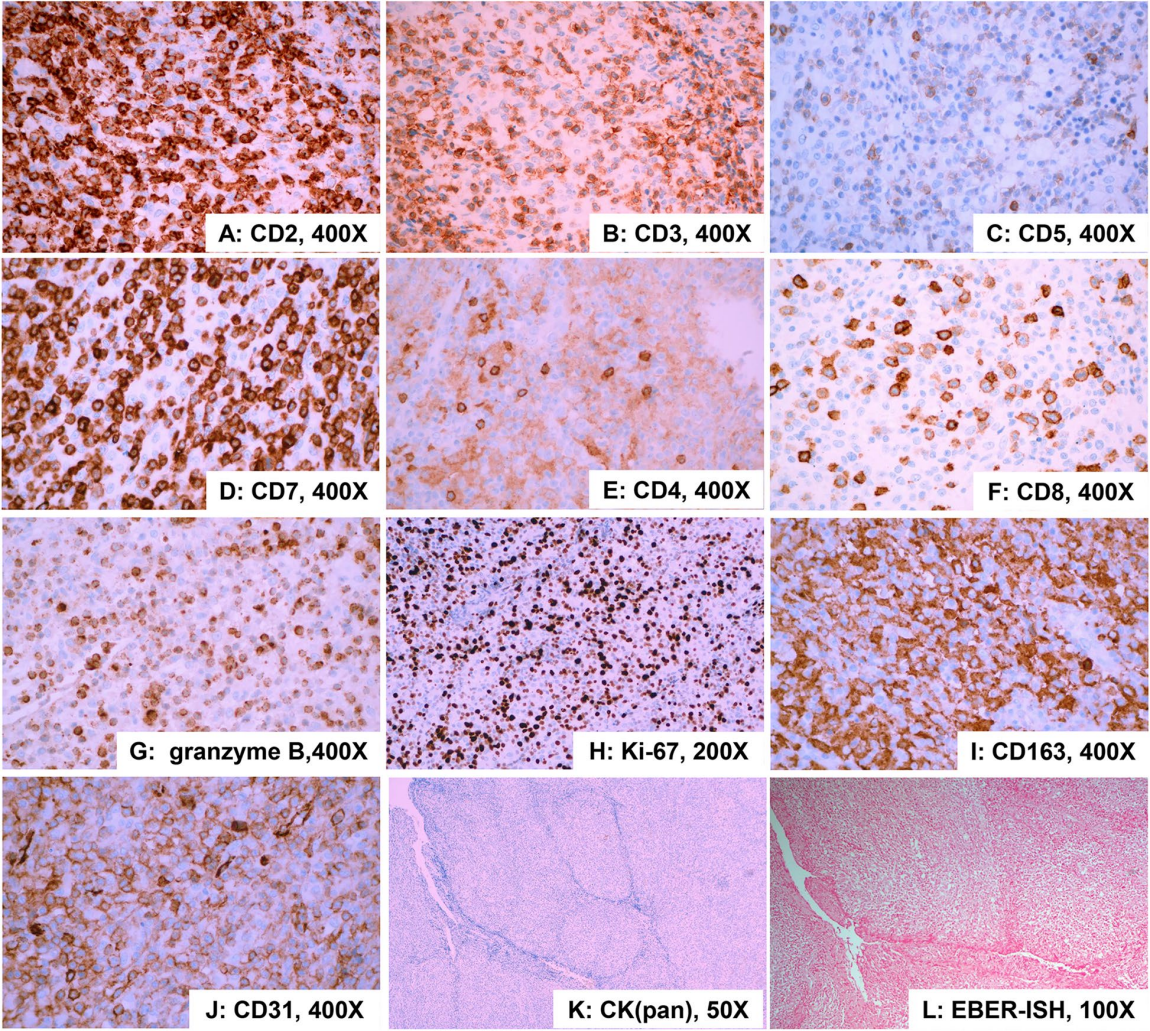
Immunohistochemical findings and EBER in situ hybridization. Immunohistochemical stains in small lymphocytes for CD2, CD3, CD5 (a few), CD7, CD4 (a few), CD8, and granzyme B are positive. Over-expressed for Ki-67. Immunohistochemical stains in epithelioid cells for CD163 and CD31 are diffusely positive, and negative for CK(pan). In situ hybridization for EBER was negative. (**A** CD2, 400×; **B** CD3, 400×; **C** CD5, 400×; **D** CD7, 400×; **E** CD4, 400×; **F** CD8, 400×; **G** granzyme B, 400×; **H** Ki-67, 200×; **I** CD163, 400×; **J** CD31, 400×; **K** CK(pan), 50×; **L** EBER-ISH, 100×)

**Fig. 4:**
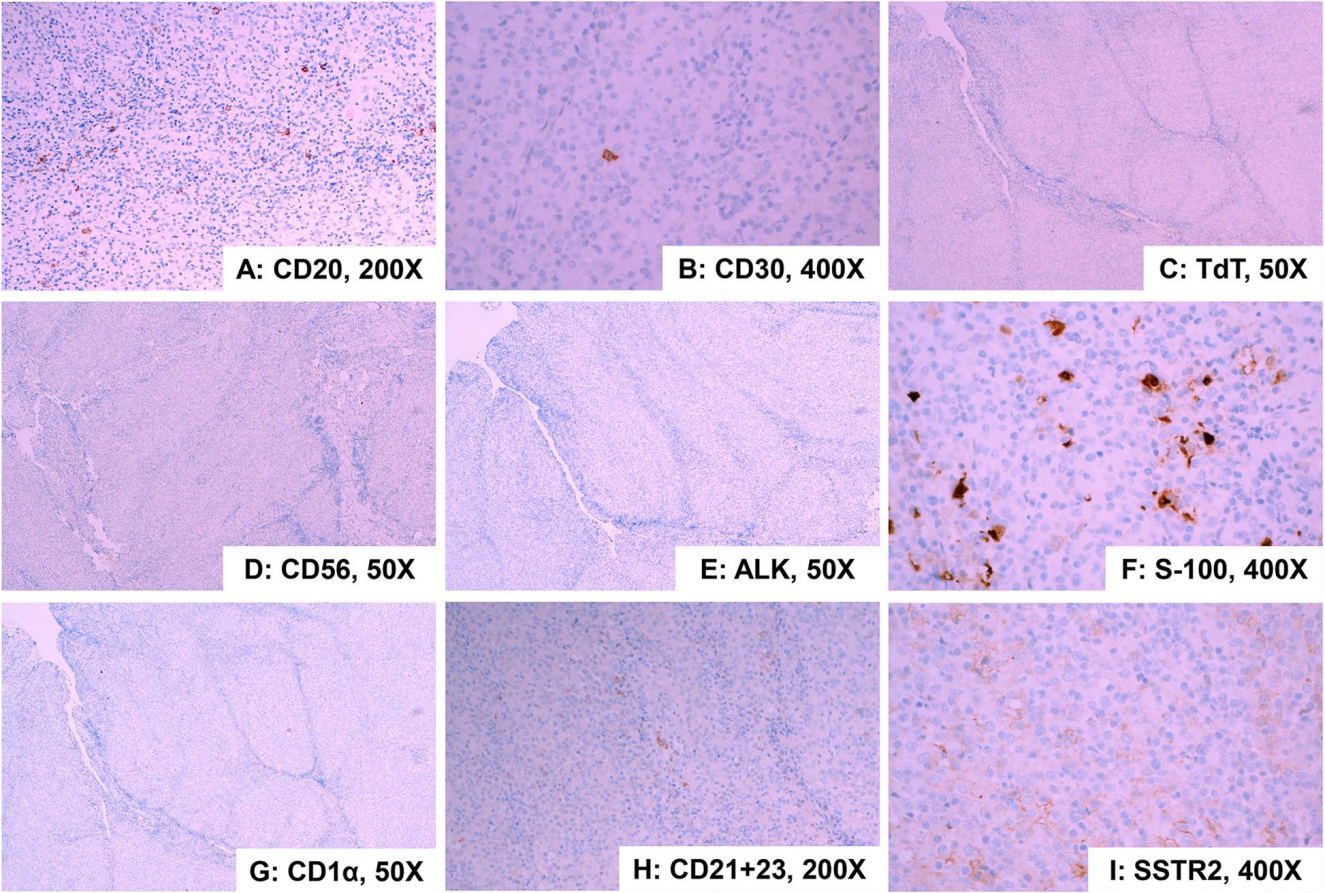
Immunohistochemical findings for other negative antibodies. Immunohistochemical stains for CD20, CD30, TdT, CD56, ALK, S-100, CD1α, CD21 + 23, and SSTR2 are negative. (**A** CD20, 200×; **B** CD30, 400×; **C** TdT, 50×; **D** CD56, 50×; **E** ALK, 50×; **F** S-100, 400×; **G** CD1α, 50×; **H** CD21 + 23, 200×; **I** SSTR2, 400×)

The imunohistochemical results showed diffuse positive expression of CD2 (Fig. 3A), CD3 (Fig. 3B), and CD7 (Fig. 3D) in atypical small lymphocytes, and significantly reduced or absent expression of CD5 (Fig. 3C). The number of CD8-positive cells (Fig. 3F) was significantly higher than CD4-positive cells (Fig. 3E). Granzyme B (Fig. 3G) staining was positive. The Ki-67 index (Fig. 3H) was elevated to 60%. In addition, diffuse positive expression of CD163 (Fig. 3I) and CD31 (Fig. 3J) in epithelioid cells was noted, and negative for CK(pan) (Fig. 3K). Epstein-Barr virus (EBV)-encoded RNA in situ hybridization (EBER-ISH) was negative (Fig. 3L). Immunohistochemical stains for CD20 (Fig. 4A), CD30 (Fig. 4B), TdT (Fig. 4C), CD56 (Fig. 4D), ALK (Fig. 4E), S-100 (Fig. 4F), CD1α (Fig. 4G), CD21 + 23 (Fig. 4H), and SSTR2 (Fig. 4I) were all negative. Taken together, the above results suggest that this case may be a lymphoma originating from a T-cell with a rare double-positive CD4^low+^/CD8^+^ immunophenotype and numerous epithelioid histiocytes.

### Molecular analysis

Polymerase chain reactions (PCRs) for detection of immunoglobulin heavy chain (IgH) hypervariable region and TCR rearrangements were performed using fresh specimens, as described previously [7]. PCRs showed a polyclonal pattern for IgH and a monoclonal TCR γ-chain rearrangement at 201.67 bp of the Vγ1F-JG1.3/2.3 interval.

Based on these findings, the final diagnosis was frontal peripheral T-cell lymphoma, not otherwise specified (PTCL/NOS), lymphoepithelioid cell variant (Lennert lymphoma).

### Treatment and follow-up

The patient received 4 cycles of cyclophosphamide, doxorubicin liposomes, vincristine, and prednisone tablets (CHOP) and was followed for 20 months. Overall treatment efficacy was achieved without lymphadenopathy, and no other discomfort or illnesses were reported.

## Discussion

LeL is is rare and special in histomorphology. Because tumor cells are limited in number and the tumor cells are small with mild atypia, misdiagnosis is common. We report a case of primary extranodal soft tissue LeL (lymphoepithelioid variant of peripheral T-cell lymphoma, unspecified) in a 65-year-old man with a rare double-positive CD4^low+^/CD8^+^ immunophenotype. This diagnosis is extremely rare and has not been reported in the literature.

LeL was first described in 1968 by Lennert et al. [8]. In 1986 the disease was first classified as “lymphoepithelioid cellular lymphoma,” and subsequently recognized as a T-cell lymphoma [8]. Patsouris et al. [4] performed immunologic studies and confirmed that LeL had a T-cell origin. It was not until 1992 that the Kiel classification officially classified LeL as a low-grade lymphoma of T-cell origin. Because the incidence of LeL is extremely low and the origin has not been clarified, LeL was classified as a peripheral T-cell lymphoma (non-specific type) by the World Health Organization Classification of Haematolymphoid Tumours [1, 2].

Lymph node histology typically reveals a destroyed lymph node follicular structure by tumor cells composed of atypical lymphocytes and epithelioid histiocytes, sometimes admixed with plasma cells, eosinophils, and Reed-Sternberg-like cells [9]. Highly endothelial small vessels are less common than other peripheral T-cell lymphomas. Necrosis has not been mentioned in any of the cases. Although lymph node involvement was not detected in this case, the histologic morphology was typical, which is basically consistent with the above characteristics.

Geissinger et al. [10] studied 101 PTCL/NOS cases, including 18 LeL cases. All cases expressed CD3, but only 94, 56, 35, 12, and 0% expressed CD5, CD2, gramB, CD30, and CD56, respectively.

The PTCL immunophenotype is categorized by CD4 and CD8. LeL, the common morphologic variant of PTCL/NOS, displays a cytotoxic T-cell phenotype of the majority of cases [2]. Bekkenk et al. [11] summarized 82 PTCL cases and found that the CD3^+^/CD4^+^/CD8^−^ was the most common type (72%), followed by CD3^+^/CD4^−^/CD8^+^ (15%), CD3^+^/CD4^−^/CD8^−^/ (11%), and CD3^+^/CD4^+^/CD8^+^ (2%). Geissinger et al. [10] studied 101 PTCL/NOS cases and showed that only 1% of cases were CD4^+^/CD8^+^, and among 18 LeL cases, no cases displayed the CD4^+^/CD8^+^ immunophenotype. The tumor cell lineage of LeL is controversial. Formerly, neoplastic cells in LeL were believed to be derived from CD4^+^ helper/inducer T cells [12]; however, it has been argued that at least part of LeL originates from cytotoxic T cells [10, 15]. Thus, the tumor cell lineage of LeL appears to be heterogenous [16]. Hartmann et al. [6] studied 18 cases of LeL and concluded that LeL is a rare and unique epithelioid cell-rich lymphoma that differs from the morphology and immunophenotype of other peripheral T-cell lymphoma subtypes, with CD8 positivity in tumor cells in the vast majority of cases. A small percentage of patients may present as CD4 ^+^ CD8^-^ or CD4^-^CD8^-^. Kim et al. [17] also reported a case of double-positive CD4/CD8 immunophenotype in LeL. In 2021, Kurita et al. [3] reported that only 1 of 26 cases exhibited double-positive CD4/CD8 immunophenotypic features (CD2^+^/CD3^+^/CD5^low+^/CD7^+^/CD4^low+^/CD8^+^/CD30^−^/CD56^−^). This finding is consistent with the immunophenotype mentioned in the literature above and is a rare immunophenotype of CD4^low+^ /CD8^+^ double-positivity.

Epstein-Barr virus (EBV) is found in approximately 30% of all PTCL-NOS cases [18]. Chihara et al. [19] reported a case of LeL complicated by monoclonal proliferation of large B-cells, and ISH of the EBV genome revealed positive signals in the nucleus of large B-lymphoid cells. Although latent EBV-infected cells are frequently present in LeL cases, the virus is probably not directly involved in the pathogenesis [20]. EBV infection may occur in a small number of immunoblasts and small B lymphocytes, as reported in LeL [20]. Kurita et al. [3] analyzed the clinicopathologic features of 26 patients with LeL and reported that 31% of the patients exhibited EBER-positive non-neoplastic cells. It has also been reported that LeL is associated with EBV+ diffuse large B-cell lymphoma [19]. A Central European single-center study showed that EBV infection might be a key factor that influences survival in LeL [21]. In the present case, EBER-ISH was negative.

LeL likely has more favorable clinical behavior compared with other types of PTCL/NOS [22]. Yamashita et al. [16] studied 10 cases of this tumor with a mean survival of 42.2 months. The shortest survival was 11 months, and the most frequent survival (still alive as of the follow-up date) was 115 months. Most of the treatments for this disease involve chemotherapy. Wanling et al. [23] were of the opinion that LeL should be regarded as a low-grade lymphoma and the intensity of chemotherapy should be appropriately increased during treatment. Stronger chemotherapy regimens should be given for initial treatment to achieve clinical remission in 1–2 courses, then stronger chemotherapy should be given to consolidate the efficacy.

Thus, this was a case of primary extranodal soft-tissue LeL, which is a rare variant of PTCL/NOS. Our case displayed a rare double-positive CD4^low+^/CD8^+^ immunophenotype. At 20 months of follow-up, the patient’s status was disease-free survival. Little is known about the clinical manifestation and prognosis of this rare PTCL type, especially the case of primary extranodal soft tissue. We report this case hoping to provide more references for clinicians and pathologists in future work to reduce missed diagnoses and misdiagnoses; however, more studies and reports are needed for this rare lymphoma.

## Data Availability

The datasets used and analysed during the current study are available from the corresponding author on reasonable request.

## Abbreviations

LeL: Lennert lymphoma
PTCL/NOS: Peripheral T-cell lymphoma-not otherwise specified
EBER-ISH: EBV-encoded RNA in situ hybridization
HRS: Hodgkin-Reed-Sternberg
IgH: Immunoglobulin heavy chain
TCR: T-cell receptor
